# Supplementary material for: Gender Differences in Anxiety, Attitudes, and Fear among Nursing Undergraduates Coping with CPR Training with PPE Kit for COVID
Source: Int J Environ Res Public Health. 2022 Nov 25;19(23):15713. doi: 10.3390/ijerph192315713 (PMC9737046; doi:10.3390/ijerph192315713)
Supplement: Supplementary file 1 [file ijerph-19-15713-s001.zip › ijerph-1991842-supplementary.pdf]

| **ID** | | | |
|---|---|---|---|
| **Age** | | **Sex** | |

The following phrases that we use to describe ourselves are given below. Read each sentence, and then check the box that best reflects what you're feeling right now, right now. Don't spend too much time, indicate the answer that best reflects your current feelings. There are no right or wrong answers.

| | **No** | **Nor if Nor not** | **Yes** |
|---|---|---|---|
| 1. I feel calm | | | |
| 2. I feel safe | | | |
| 3. I feel nervous | | | |
| 4. I feel scared | | | |
| 5. I feel happy | | | |
| 6. I feel at ease | | | |
| 7. I feel relaxed | | | |
| 8. I feel worried | | | |
| 9. In general, do you feel emotionally prepared to perform quality CPR? | | | |
| 10. Have you ever worn personal protective equipment type 4, 5 or 6? | | | |

| 11. How do you feel about performing CPR under conditions of possible biological risk or pandemic? (Check one or several) | | |
|---|---|---|
| Nothing, no hassle<br>Anxiety<br>Displeasure | Curiosity<br>Uncertainty<br>Fear | Else: |

| 12. What do you think worries you most when performing CP R with PPE? (Check one or several) | | |
|---|---|---|
| Nothing, no hassle<br>That the patient dies | Possible contagion<br>Do not do CPR of quality | Else: |

| | | | |
|---|---|---|---|
| **Name and Surnames** | | | |
| **Age** | | **Sex** | |

The following phrases that we use to describe ourselves are given below. Read each sentence, and then check the box that best reflects what you're feeling right now, right now. Don't spend too much time, indicate the answer that best reflects your current feelings. There are no right or wrong answers.

| | **No** | **Nor if Nor not** | **Yes** |
|---|---|---|---|
| 1. I feel calm | | | |
| 2. I feel safe | | | |
| 3. I feel nervous | | | |
| 4. I feel scared | | | |
| 5. I feel happy | | | |
| 6. I feel at ease | | | |
| 7. I feel relaxed | | | |
| 8. I feel worried | | | |
| 9. In general, have you felt emotionally prepared to perform quality CPR? | | | |
| 10. Have you had thoughts about life and death when you had quality CPR? | | | |
| 11. Have you been afraid of losing control during CPR? (Leaving the room, feeling sick or dizzy, etc.) | | | |
| 12. After practice, do you think more quality and concentration training would be needed to complete quality CPR? | | | |
| 13 Do you think it is necessary to carry out more CPR practices during the 4 years of Nursing? | | | |
| 14. I would recommend this practice for other courses | | | |

Say how **satisfied** you are with the practice you have done today (check one):

□ very dissatisfied

□ Unsatisfied

□ Satisfied

□ Very satisfied

| | |
|---|---|
| **How would you rate your overall satisfaction with the practice? (From 0 to 10, where 0 is the worst grade, and 10 is the best grade?** | |
